# Refracture after implant removal of the clavicle: a retrospective cohort analysis

**DOI:** 10.1186/s13018-026-07123-5

**Published:** 2026-07-21

**Authors:** Ahmed Ellafi, Eva Coenen, Tobias Resch, Philipp Zehnder, Michael Zyskowski, Lukas Willinger, Peter Biberthaler, Markus Schwarz

**Affiliations:** 1 Department of Trauma Surgery, TUM University Hospital Rechts der Isar, Technical University of Munich, Ismaninger Str. 22, 81675 Munich, Germany; 2 Department of Sports Orthopedics, TUM University Hospital Rechts der Isar, Technical University of Munich, Ismaninger Str. 22, 81675 Munich, Germany

**Keywords:** Clavicle fracture, Refracture, Implant removal, Plate osteosynthesis, Risk factors

## Abstract

**Background:**

Refracture after implant removal in clavicle fractures is a relevant but insufficiently studied complication. Reported refracture rates vary due to small cohorts and heterogeneous populations. This study aims to determine the incidence of refracture following elective implant removal after confirmed fracture union and to identify potential demographic, clinical, and fracture-related risk factors.

**Methods:**

A retrospective cohort of 575 adults who underwent implant removal after radiographically confirmed union between 2011 and 2024 was analyzed. Patients were assigned to a refracture (R) or non-refracture (NR) group. Demographics, fracture characteristics, treatment variables, and time intervals were analyzed.

**Results:**

Refractures occurred in 21/575 patients (3.7%). No significant differences were observed between the R and NR groups regarding age, BMI, ASA classification, or tobacco use. The interval between initial fixation and implant removal was shorter in the refracture group but not statistically significant (18.8 ± 8.7 vs. 21.2 ± 18.5 months; *p* = 0.55). Most refractures occurred at the original fracture site (n = 19; 90.5%) and were mostly midshaft fractures (n = 18; 85.7%). Only the AO 15.2C fracture type showed a significant association with refracture (R: 11.1% vs. NR: 1.9%; p = 0.005). In time-to-event analysis, AO 15.2C fractures were independently associated with refracture (HR 6.70, 95% CI 1.49–30.12; *p* = 0.013). Refracture-free survival was 97.4% at 1 year and 96.2% at 10 years. Implant removal was most frequently performed due to patient preference (R: 66.7% vs. NR: 50%).

**Conclusion:**

Refracture after clavicle implant removal is an uncommon yet clinically relevant complication. The overall refracture rate was 3.7% in the present study. Neither demographic variables nor implant retention time were significantly associated with refracture risk, whereas fracture morphology—specifically complex midshaft fractures (AO 15.2C)—was the only significant risk factor identified. Fracture morphology should therefore be a key consideration when deciding on elective implant removal. Further prospective research is needed to refine guidelines on optimal timing and patient selection.

## Introduction

Clavicle fractures are common injuries, accounting for roughly 2.6–4% of all fractures in adults [1]. About 80% of these fractures involve the middle third of the clavicle [2, 3]. Historically, the majority of clavicle fractures were managed nonoperatively [4]. The last two decades have highlighted elevated non-union rates and inferior shoulder outcomes with nonsurgical treatment of displaced midshaft injuries, prompting increased use of operative therapy [5–8]. The current surgical gold standard for the treatment of displaced midshaft clavicle fractures is open reduction and internal fixation (ORIF) using locking plate osteosynthesis [9]. Lateral fractures may be managed with a hook plate, anatomic locking plate, or minimally invasive acromioclavicular joint reconstruction [10, 11]. Studies have shown that surgical management of clavicle fractures enables earlier mobilization and superior functional outcomes compared with nonoperative care [8, 12]. Despite high union rates and good functional outcomes, implant-related discomfort is common [13]. Due to minimal soft tissue coverage and the prominence of superiorly placed plates, implants may cause local irritation and tenderness [13]. As a result, many patients request elective implant removal after the fracture has healed. Studies have reported that about 38–50% to over 70% of patients undergo implant removal following clavicle ORIF, depending on the patient population and local practice [14, 15]. The most common reasons for requesting implant removal are skin irritation or palpable implant, pain or stiffness around the shoulder, and sometimes cosmetic concerns [13]. It is important to note that routine plate removal is not generally recommended unless there are symptoms or other specific indications [16]. Removing a plate entails another surgery with its own risks, and some patients may not experience any benefit if their only complaint is vague discomfort [17, 18]. Current practice is to leave the clavicle plate in place if it is not causing problems, or at least until a sufficient time has passed for complete bony remodelling, and to carefully weigh the risks and benefits before implant removal [16]. When performed, implant removal in the upper extremity is typically delayed until fracture union is confirmed, often after 12–18 months [17, 18].

One serious complication following implant removal is refracture of the clavicle. Fortunately, this does not happen in the majority of patients, but the incidence is notable enough to warrant caution. Studies have reported refracture rates ranging roughly from 2% up to 7% of cases [13, 19–22]. Although infrequent, refractures represent a clinically relevant complication that may require additional treatment [21].

However, the existing literature is constrained by small cohorts and few refracture events, limiting the precision and generalizability of reported rates. To address this gap, the present study analyses a larger patient cohort and undertakes a systematic evaluation of refracture incidence, timing, and independent risk factors. Therefore, the aim of the present study was to determine the incidence and timing of refracture after elective clavicle implant removal following confirmed fracture union and to identify potential demographic, clinical, and fracture-related risk factors associated with refracture occurrence.

## Methods

### Setting and study design

This retrospective cohort study was conducted at TUM Klinikum Rechts der Isar, Technical University of Munich, a German level 1 trauma centre. Ethical approval was obtained from the institutional review board, and the study was performed in accordance with the Declaration of Helsinki (Ethics Committee of the Technical University of Munich, Germany, 2020-2_1-S-SB).

### Study population

The institutional clinical information system was screened to identify all adult patients (aged ≥ 18 years) who underwent operative treatment for clavicle fractures between January 2011 and December 2024 (n = 1326). From this cohort, we included all patients who subsequently underwent elective implant removal after plate osteosynthesis once radiographic bone union had been confirmed (Implant removal cohort: n = 575).

Exclusion criteria comprised peri-implant clavicle fractures, pathological fractures, and incomplete clinical or radiographic documentation. Indications for implant removal included patient-reported symptoms (implant prominence or irritation, weather-dependent pain, restricted shoulder motion) or patient preference after confirmed fracture consolidation. Radiographic fracture consolidation prior to implant removal was assessed during routine outpatient follow-up using standard anteroposterior and tangential clavicle radiographs. Fracture consolidation was determined by the treating orthopaedic trauma surgeons based on cortical bridging, absence of secondary displacement, and clinical absence of local tenderness or instability. Implant removal was only considered after confirmed fracture consolidation. Demographic data and comorbidities were extracted from the initial clinical records. Fractures were classified according to the AO/OTA classification (AO Foundation/Orthopaedic Trauma Association Classification) for midshaft fractures [23], the Throckmorton classification for medial fractures [24], and the Jäger and Breitner classification for lateral fractures [25]. In case of plate osteosynthesis, superior plate fixation was the standard technique at our institution; therefore, no comparison regarding the plate position was possible.

### Statistical analysis

Patients were categorized into a refracture (R) and a non-refracture (NR) group for descriptive comparisons. Continuous variables were assessed for normal distribution and are presented as mean ± standard deviation (SD). Group comparisons were performed using independent t-tests for continuous variables and χ^2^-tests or Fisher’s exact tests for categorical variables, as appropriate. Refracture-free survival following implant removal was analysed using the Kaplan–Meier method. Cumulative survival rates were calculated at 1, 2, 5, and 10 years. Differences between groups were assessed using the log-rank test.

To identify independent predictors of refracture, a multivariable Cox proportional hazards regression model was constructed. To avoid overfitting, the number of variables included in the model was restricted. Variables were selected based on clinical relevance, findings from the existing literature, and results of the univariable analyses. The final model included AO 15.2C fracture morphology, implant retention time, and BMI. Hazard ratios (HR) with 95% confidence intervals (CI) were reported. A *p*-value < 0.05 was considered statistically significant. Statistical analyses were performed using IBM SPSS Statistics (Version 29.0; IBM Corp., Armonk, NY, USA) and Microsoft Excel (Version 16.98).

## Results

Among the 1326 patients who underwent surgical treatment for clavicle fractures, 575 individuals (43.4%) who underwent implant removal after radiologically confirmed bone union were included in this study. The cohort consisted of 433 men (75.3%) and 142 women (24.7%), all aged 18 years or older. The mean age was 39.2 ± 14.0 years, the mean body mass index (BMI) was 23.6 ± 3.3 kg/m^2^, the mean body weight was 75.1 ± 13.7 kg, and the mean height was 178.0 ± 8.7 cm. The mean interval between the initial fixation and implant removal was 21.2 ± 18.0 months. The median interval between initial fixation and implant removal was 19.4 months (IQR 14.1–24.8 months; range 3.6–152.9 months).

All patients were subsequently divided into two groups: a refracture group (n = 21; 3.7%) consisting of individuals who sustained a refracture after implant removal, and a non-refracture group (n = 554; 96.3%) with uneventful healing following implant removal.

### Patient characteristics

Baseline demographic and clinical characteristics are summarized in Table 1.

**Table 1:** Patient characteristics of the refracture and non-refracture groups

Variable	Refractures (n = 21)	No refractures (n = 554)	*p*-value
Female n (%)	7 (33.3)	135 (24.4)	0.35
Male n (%)	14 (66.7)	419 (75.6)	
Age mean ± SD years	35.4 ± 15.9	39.4 ± 13.3	0.18
Weight mean ± SD kg	73.9 ± 16.6	75.1 ± 13.2	0.68
Height mean ± SD cm	177.3 ± 10	178.0 ± 8.6	0.70
BMI mean ± SD kg/m^2^	23.3 ± 3.8	23.6 ± 3.3	0.69
ASA n (%)			0.66
I	14 (66.7)	395 (71.3)	
II	6 (28.6)	153 (27.6)	
III	1 (4.8)	6 (1.1)	
Tobacco use n (%)	6 (28.6)	157 (28.3)	0.98
Interval between ORIF and implant removal mean ± SD months	18.8 ± 8.7	21.2 ± 18.5	0.55
Main reason for implant removal n (%)			
Patient preference	14 (66.7)	277 (50)	
Pain or irritation	5 (23.8)	209 (37.7)	
Other	2 (9.5)	68 (12.3)	

ASA: American Society of Anesthesiologists Classification; BMI: Body mass index; ORIF: Open reduction and internal fixation; SD: Standard deviation

No significant differences between the refracture (R) and non-refracture (NR) groups were observed regarding age, sex, BMI, ASA classification, tobacco use, or implant retention time (all *p* > 0.05). Patient preference represented the most common indication for implant removal in both groups.

### Refracture group (R)

**Fig. 1:**
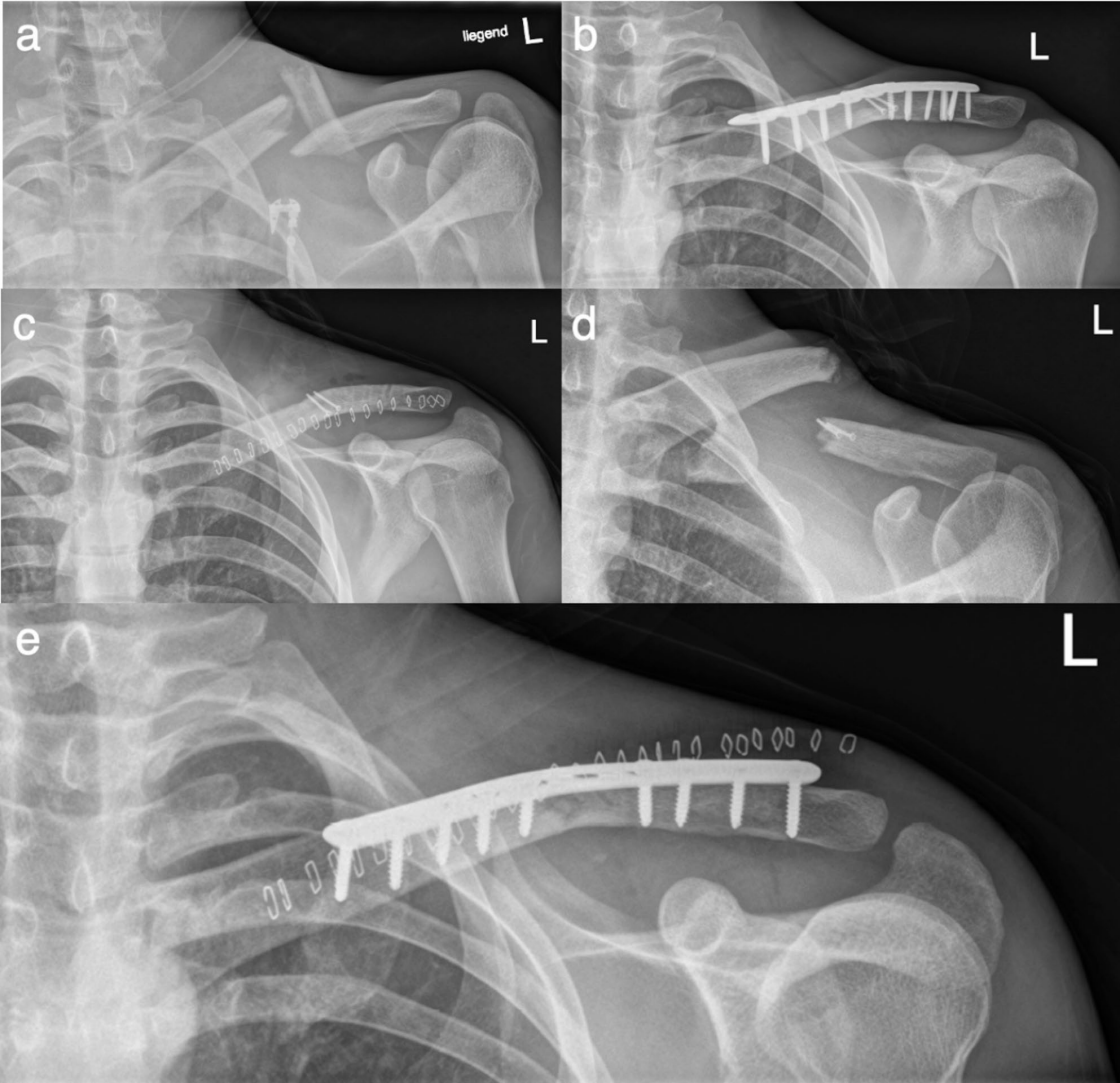
Midshaft clavicle fracture (AO 15.2C). **a** Preoperative radiograph (AP view); **b** postoperative radiograph after osteosynthesis using a locking plate with two interfragmentary lag screws (AP view); **c** radiograph after implant removal with the interfragmentary lag screws left in place (AP view); **d** refracture at the original fracture site (AP view); **e** postoperative radiograph after revision surgery using a locking plate (AP view)

A total of 21 patients (3.7%) sustained a refracture after implant removal. Refractures occurred at a mean of 15.8 ± 25.3 months after implant removal and were predominantly associated with a new traumatic event (71.4%). The median interval between initial fixation and implant removal in the refracture group was 19.2 months (IQR 11.1–24.5 months; range 3.6–32.0 months). Most refractures involved the midshaft clavicle and occurred at the original fracture site (90.5%) (Fig. 1). All refractures were treated operatively with plate osteosynthesis. Further characteristics of refracture cases are summarized in Table 2.

**Table 2:** Characteristics of refracture cases (n = 21)

Variable	N (%)/mean ± SD
Mechanism	
Traumatic	15 (71.4)
Non-traumatic	6 (28.6)
Time from implant removal to refracture (months)	15.8 ± 25.3
Refracture location	
Same site as primary fracture	19 (90.5)
Previous screw hole	1 (4.8)
Different location	1 (4.8)
Treatment characteristics after refracture	
Locking plate use	21 (100)
Interfragmentary screws used	3 (14.3)
Iliac crest cancellous bone graft used	6 (28.6)
Titanium elastic nailing	0 (0)

SD: standard deviation

### Non-refracture group (NR)

The non-refracture group consisted of 554 patients. Most fractures involved the midshaft clavicle (73.8%), followed by lateral fractures (25.3%), whereas medial fractures were rare (0.9%). Fracture classifications and treatment characteristics are summarized in Table 3.

**Table 3:** Fracture and treatment characteristics

Fracture location/variable	Refractures (n = 21) N (%)	No refractures (n = 554) N (%)	*p*-Value
Medial	0 (0.0)	5 (0.9)	1.00
Classification Throckmorton			
A	0 (0.0)	4 (80.0)	
B	0 (0.0)	1 (20.0)	
C	0 (0.0)	0 (0.0)	
Treatment characteristics			
Locking plate	0 (0.0)	4 (80.0)	
Interfragmentary lag screws	0 (0.0)	1 (20.0)	
Titanium elastic nailing	0 (0.0)	1 (20.0)	
Shaft	18 (85.7)	409 (73.8)	0.22
Classification AO			0.024*
15.2A	8 (44.4)	146 (35.7)	0.23
15.2B	8 (44.4)	255 (62.3)	0.48
15.2C	2 (11.1)	8 (1.9)	0.005*
Treatment characteristics			
Locking plate	14 (77.8)	351 (85.8)	
Titanium elastic nailing	4 (22.2)	58 (14.2)	0.2
Interfragmentary lag screws	4 (22.2)	203 (49.6)	0.1
Lateral	3 (14.3)	140 (25.3)	0.25
Classification J and B			*0.53*
I	0 (0.0)	16 (11.4)	
IIa	2 (66.7)	73 (52.1)	0.63
IIb	0 (0.0)	34 (24.3)	
III	1 (33.3)	17 (12.1)	0.66
Treatment characteristics			
Locking plate	3 (100.0)	139 (99.3)	
DogBone™	1 (33.3)	71 (50.7)	0.27
Interfragmentary lag screws	1 (33.3)	25 (17.9)	0.96

*Statistically significant; AO: AO foundation/orthopedic trauma association; J and B: Jäger and Breitner

When comparing both groups, medial fractures showed no association with refracture. Likewise, the overall frequency of midshaft fractures did not differ significantly between groups (*p* = 0.22). Among midshaft fractures, AO types 15.2A and 15.2B demonstrated no significant differences. However, the two groups differed significantly in their overall AO fracture classification distribution (*p* = 0.024), driven by the markedly higher proportion of AO 15.2C fractures in the refracture group (11.1% vs. 1.9%; *p* = 0.005). AO 15.2C was the only fracture subtype that showed a significant association with an increased risk of refracture.

Other treatment variables showed no significant associations with refracture occurrence. Similarly, neither the distribution of Jäger and Breitner fracture types nor lateral fracture treatment strategies differed significantly between groups (all *p* > 0.05).

In summary, the only statistically significant predictor which could be identified for refractures was the presence of a complex segmental midshaft fracture (AO 15.2C). All other fracture types, classifications, and treatment parameters demonstrated no significant association with refracture risk.

### Refracture-free survival analysis

Kaplan–Meier analysis demonstrated a refracture-free survival of 97.4% (95% CI, 96.0–98.8%) at 1 year, 97.2% (95% CI, 95.8–98.6%) at 2 years, 96.7% (95% CI, 95.1–98.3%) at 5 years, and 96.2% (95% CI, 94.6–97.8%) following implant removal. Most refractures occurred within the first two years after implant removal, with the survival curves plateauing thereafter. The numbers of patients at risk were 560 at 1 year, 556 at 2 years, 476 at 5 years, and 455 at 10 years after implant removal.

When stratified by fracture morphology, patients with AO 15.2C fractures showed significantly lower refracture-free survival compared with other fracture types (log-rank *p* = 0.01).

**Fig. 2:**
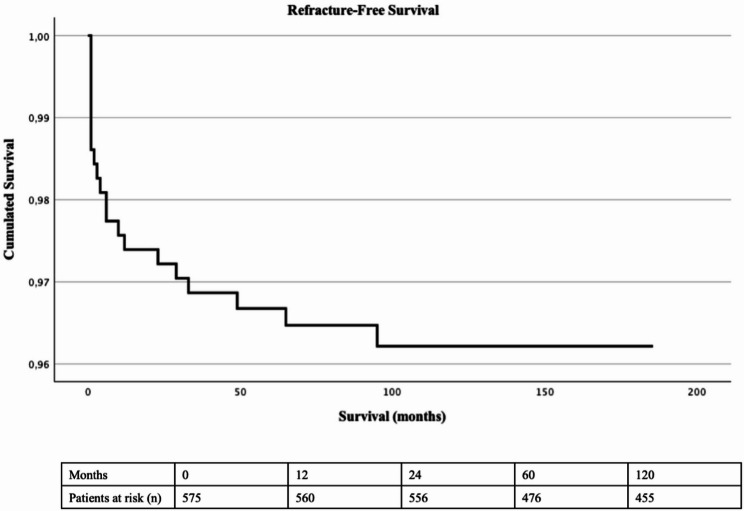
Kaplan–Meier curve showing refracture-free survival after clavicle implant removal. The numbers of patients at risk at selected time points are displayed below the x-axis

In multivariable Cox proportional hazards regression analysis, AO 15.2C fractures were independently associated with an increased hazard of refracture (HR 6.70, 95% CI 1.49–30.12; *p* = 0.013). Implant retention time (HR 0.97 per month, 95% CI 0.94–1.01; *p* = 0.119) and BMI (HR 0.98, 95% CI 0.86–1.12; *p* = 0.781) were not significantly associated with refracture risk (Fig. 2).

## Discussion

In this retrospective single-centre study of 575 patients who underwent elective implant removal after operative treatment of a clavicle fracture, the overall refracture rate was 3.7%. Most refractures occurred within the first two years after implant removal. While demographic factors, comorbidities, and implant retention time were not associated with refracture risk, complex midshaft fractures (AO 15.2C) were identified as the only independent risk factor. Thus, this work represents the largest study to date on refractures following implant removal of the clavicle. The observed refracture rate is consistent with previous reports, which range from approximately 2% to over 7% [13, 19–22]. Refractures after implant removal are therefore relatively rare but clinically relevant complications that must be taken into account when considering the indication for implant removal.

Regarding demographic factors, a previous study identified female sex and low BMI as independent risk factors for refracture after implant removal [21]. In contrast, the presented analysis did not demonstrate any significant association between the occurrence of refracture and demographic parameters such as age, sex, body weight, height, BMI, ASA score, or tobacco use. These findings are consistent with several more recent studies that likewise failed to confirm any sex- or BMI-related effects [19, 20, 26].

A central finding of this study is the significant difference in fracture morphology between patients with and without refracture. The overall distribution of the AO/OTA classification differed significantly between the two groups, indicating that the type of the initial fracture has a substantial impact on refracture risk. A similar difference has been reported in previous studies, which supports the presented findings [17, 20]. Notably, complex midshaft fractures of the AO type 15.2C occurred markedly more often in the refracture group and represented a statistically significant risk factor. While the much more common fracture types 15.2A and 15.2B did not differ between groups, type 15.2C was clearly associated with an increased risk of refracture after implant removal. This observation is clinically plausible, as AO 15.2C fractures are characterized by greater comminution and displacement, often associated with reduced fragment contact and local bone loss. These factors may contribute to delayed remodelling and persistent stress concentration at the former fracture site, which may explain the higher refracture rate observed in this subgroup. This observation is consistent with previous literature showing that highly displaced and multifragmentary fractures are associated with less favourable healing outcomes [27, 28]. Regarding the location of refractures, the vast majority occurred at the original fracture site, while refractures originating from former screw holes were observed only rarely. This pattern is consistent with previous studies, which likewise reported a clear clustering of refractures at the primary fracture zone and suggest that local structural weakness may persist despite radiographic evidence of union [21, 22].

Most refractures in our cohort were associated with a new traumatic event. This suggests that refracture is not only a spontaneous biological failure but often occurs when new trauma affects a still vulnerable fracture site. At the same time, the predominance of refractures at the original fracture site indicates that local structural weakness may persist even after radiographic union. The assessment of fracture healing prior to implant removal represents an important aspect when interpreting these findings. In the present study, fracture union was primarily confirmed using plain radiographs, which reflects routine clinical practice but may not reliably detect incomplete cortical bridging or asymmetric healing. Therefore, residual biological or mechanical vulnerability may have remained undetected in some patients despite apparent radiographic union. This may further contribute to the observed clustering of refractures at the original fracture site.

Implant retention time was not significantly associated with refracture risk in the present cohort. Although a trend toward a lower hazard with longer implant retention was observed, this did not reach statistical significance and should therefore be interpreted with caution. The Kaplan–Meier analysis demonstrated that most refractures occurred within the first two years after implant removal, with survival curves plateauing thereafter. This suggests that the period immediately following implant removal represents the highest vulnerability window. The influence of implant time in situ on the risk of refracture is inconsistently described throughout the literature. Some studies report no association between the duration of implant retention and the risk of refracture, whereas others indicate that both very early and very late implant removal may be associated with increased vulnerability [17, 21]. For example, one study found no significant effect of time in situ, even though implant removal was typically performed approximately one year after the initial osteosynthesis [21]. In contrast, other articles have shown that implant removal within the first 12–18 months—particularly in forearm fractures—may increase the risk of refracture [13, 17], whereas prolonged implant retention beyond 2–3 years in proximal femur fractures has likewise been associated with higher complication rates, including refracture [13, 29]. However, due to the substantially different biomechanical loading conditions of the femur as a primary weight-bearing bone, these findings are only of limited comparability to the clavicle. These divergent findings indicate that no clearly defined optimal time window for implant removal currently exists and that both “too early” and “too late” removal may carry potential risks. In our cohort, implant retention time was not identified as an independent risk factor, suggesting that timing alone may not be a decisive determinant of refracture risk.

In this cohort, the most common indication for implant removal was patients’ preference, followed by local irritation or pain, which is consistent with previously published data [18, 30]. Several studies emphasize that subjective discomfort is a frequent but often unspecific indication, and postoperative symptom relief in these cases is not guaranteed [30]. Although neurological symptoms may also contribute to implant-related complaints after clavicle fixation, no documented neuroma was identified in our cohort. Dysesthesia occurred only transiently and resolved during follow-up, and no persistent supraclavicular nerve palsy or neuropathic pain requiring nerve-related procedures was documented. Given the observed refracture risk of 3.7%, the indication for elective implant removal should therefore be made cautiously, especially when symptoms are mild or non-specific.

## Limitations

This study has several limitations. First, despite the overall large cohort, the absolute number of refracture cases was small (n = 21), which limits the statistical power for subgroup analyses and the detection of additional risk factors. The limited number of events also restricts the complexity of multivariable time-to-event modelling and may reduce the precision of hazard ratio estimates, as reflected by the relatively wide confidence intervals. Given the low number of refracture events, the results of the multivariable Cox regression should be interpreted with caution due to the potential risk of overfitting. Second, fracture healing prior to implant removal was primarily assessed using standard radiographs; CT imaging was not routinely performed, which may have limited the detection of incomplete or asymmetric union. Furthermore, despite radiographic confirmation of fracture union, implant removal was performed according to patient symptoms or preference; therefore, a degree of selection bias cannot be excluded. In addition, incision length, platysma incision, and the degree of anterior soft tissue coverage were not systematically documented and therefore could not be evaluated retrospectively. Finally, follow-up completeness was not formally assessed, and refractures treated at other institutions may therefore have been missed, potentially resulting in an underestimation of the true refracture incidence. Nevertheless, the use of Kaplan–Meier survival estimates and multivariable Cox regression allowed for a time-adjusted risk assessment and strengthens the robustness of the present findings despite the low event rate.

## Conclusion

Refractures after implant removal of the clavicle are uncommon but clinically relevant complications. In this large cohort, demographic factors and implant retention time showed no association with refracture risk, whereas complex midshaft fractures—particularly AO type 15.2C—were found to be a significant risk factor. These findings highlight the importance of considering fracture morphology when evaluating patients for elective implant removal. Careful patient selection and thorough counselling remain essential to minimize postoperative complication rates. Future studies with larger sample sizes and prospective designs are required to clarify the impact of implant removal timing on refracture risk.

## Data Availability

The datasets generated and analyzed during the current study are available from the corresponding author on reasonable request.

## Abbreviations

AO/OTA: AO foundation/orthopaedic trauma association classification
ASA: American society of anesthesiologists classification
BMI: Body mass index
CI: Confidence interval
CT: Computed tomography
HR: Hazard ratio
NR: Non refracture
ORIF: Open reduction and internal fixation
R: Refracture
SD: Standard deviation
